# Insights into the role of mesenchymal stem cells in cutaneous medical aesthetics: from basics to clinics

**DOI:** 10.1186/s13287-024-03774-5

**Published:** 2024-06-18

**Authors:** Junyi Li, Ye Liu, Rui Zhang, Qianyu Yang, Wei Xiong, Yan He, Qingsong Ye

**Affiliations:** 1 Center of Regenerative Medicine, Renmin Hospital of Wuhan University, Wuhan, 430060 China; 2 Institute of Regenerative and Translational Medicine, Tianyou Hospital, Wuhan University of Science and Technology, Wuhan, 430030 China

**Keywords:** Mesenchymal stem cells, Cutaneous medical aesthetics, Wound healing, Scar repair, Skin rejuvenation

## Abstract

With the development of the economy and the increasing prevalence of skin problems, cutaneous medical aesthetics are gaining more and more attention. Skin disorders like poor wound healing, aging, and pigmentation have an impact not only on appearance but also on patients with physical and psychological issues, and even impose a significant financial burden on families and society. However, due to the complexities of its occurrence, present treatment options cannot produce optimal outcomes, indicating a dire need for new and effective treatments. Mesenchymal stem cells (MSCs) and their secretomics treatment is a new regenerative medicine therapy that promotes and regulates endogenous stem cell populations and/or replenishes cell pools to achieve tissue homeostasis and regeneration. It has demonstrated remarkable advantages in several skin-related in vivo and in vitro investigations, aiding in the improvement of skin conditions and the promotion of skin aesthetics. As a result, this review gives a complete description of recent scientific breakthroughs in MSCs for skin aesthetics and the limitations of their clinical applications, aiming to provide new ideas for future research and clinical transformation.

## Background

The skin, the biggest organ in the human body, serves several vital physiological and biological functions in addition to its aesthetic value, such as protecting the body from harmful substances, assisting in the perception of diverse sensations, and regulating temperature [1, 2]. Skin problems not only reduce patients’ quality of life and create psychological strain, but also bring a heavy economic burden to families and society [3–5]. Many skin diseases are associated with adult depression [6, 7]. Human skin wounds cause a significant epidemiological and financial cost. With the aging of the population and the increase of known comorbidity incidence rate affecting wound healing, its impact will continue to increase [8]. Hypopigmentation can cause aesthetic and psychological problems, reducing patients’ quality of life [9]. Scar is a common phenomenon after wound healing that seriously affects the appearance of the wound and brings about aesthetic, functional, and/or psychological issues [10, 11]. Especially facial scars are more likely to lead to functional defects and psychological burden. The market for scar therapy is anticipated to grow to around 32 billion US dollars by 2027 [12].

Therefore, skin aesthetics have become increasingly fashionable in recent years as the economy has grown. Internal aging and the stimulation of numerous external irritants ultimately affect the skin structure, resulting in cosmetic issues such as wrinkles and hair loss as well as functional issues such as barrier maintenance hurdles [13]. The internal aging mechanism of the skin is complex, including the accumulation of gene mutations, DNA damage, cellular aging, inflammation, and oxidative stress (OS) [14, 15]. External aging is the consequence of a combination of environmental causes, including ultraviolet (UV) light, PM2.5, nitrogen dioxide, ground ozone, cigarette smoke, food additives, and heavy metal ions, ultimately leading to DNA damage and cellular dysfunction [15, 16]. This has a significant influence on the lives of patients, resulting in a variety of physiological and psychological issues. As a result, individuals are increasingly looking for effective and safe medical cosmetic treatments to tackle skin concerns. There are many ways to improve the skin condition, such as through skin care, medications, laser, and surgery [17]. However, each of these approaches has its own set of drawbacks and fails to produce the desired results in terms of skin repair and regeneration [18]. As a current hot research topic, by promoting and controlling endogenous stem cell populations and/or restocking cell pools for organizational stability and regeneration, stem cell-based treatments constitute a crucial subspecialty of regenerative medicine and have achieved superior therapeutic effects [19].

Stem cells possess advantageous characteristics, like being able to self-regenerate and specialize into several cell types. Stem cells alone, stem cell secretion groups, and stem cells combined with nanomaterials are the three major ways that stem cell treatment is now applied [20]. They have been extensively investigated in treatments to treat numerous human maladies, including Type 1 diabetes, Alzheimer’s disease, Parkinson’s disease, spinal cord injury, and cancer [21–25]. Yet there are certain risks to stem cell treatment that cannot be overlooked, such as genomic instability during cell expansion, cell malignancy, the possibility of increased tumor development in vivo, and the possibility of poor cell differentiation [26–28]. There are three types of stem cells employed for therapeutic purposes: embryonic stem cells (ESCs), induced pluripotent stem cells (iPSCs), and adult stem cells like MSCs [29]. ESCs, originating from embryos’ inner cell aggregate, possess pluripotent properties and hold the potential to differentiate into a full range of cell types. However, there are major restrictions on using ESCs in clinical practice due to ethical issues [30]. The essence of ethical issues in ESCs is that obtaining ESCs requires the devastation of early embryos, which are considered to have the moral status of a complete human and possess enormous moral sanctity. Therefore, it is not morally permissible to use them for scientific research or therapeutic purposes [31–33]. The potential alternatives for ESCs are iPSCs and MSCs [34]. IPSCs may be created from mature cells by gene editing and ectopic expression of particular pluripotent stem factors, thus avoiding many ethical issues [35]. However, there are still challenges in the process of creating iPSCs, such as monitoring and reducing the genetic instability of iPSCs and enhancing immune compatibility [36, 37]. And due to genetic instability, iPSCs have tumorigenic potential [34, 36]. Therefore, further study is required to develop a reliable, repeatable, and successful reprogramming strategy [38]. MSCs can be derived from various tissues. Most studies agree that these adult stem cells are abundant, diverse in origin, easy to harvest and isolate, have strong pluripotent differentiation ability, and therefore have multiple applications [39–42]. Recent research has revealed that MSCs can promote skin wound healing, pigmentation modulation, and anti-aging as a therapeutic option for cutaneous medical aesthetics [43–46].

Currently, research on the combined use of MSCs and nanomaterials focuses on employing materials to create an environment that favors cell survival, differentiation, proliferation, and paracrine secretion, promoting the greater efficacy of MSCs [47, 48]. Although these nanomaterials have achieved good preclinical efficacy, biocompatibility issues, immune issues, and mechanical properties still need improvement [49].

We systematically searched PubMed and Web of Science for papers related to mesenchymal stem cells, dermatological aesthetics, wound healing, scar repair, skin rejuvenation, and anti-pigmentation from 1975 to February 2024. Here, we will describe the most current findings on the processes and uses of MSCs and their secretomics in skin medical aesthetics, such as wound healing, scar repair, skin rejuvenation, and pigmentation modification. A deeper knowledge of their respective roles will clarify the use of stem cell therapy in cutaneous medical aesthetics, providing new strategies for the future.

## Mechanisms underlying skin damage

### The basis of the skin physiology

The skin, comprising the epidermis, dermis, and subcutaneous tissues, serves as the human body’s biggest biological, chemical, and immunological barrier [2, 50]. The cuticle, which is the skin’s exterior layer, measures 10–20 μm thick and is composed of 10–15 layers of interconnected dead cells. The subsequent layer, known as the living epidermis, has a thickness of 100–150 μm and primarily comprises keratin-producing cells in various stages of differentiation [51]. The third layer, the dermis, is abundant in growth factors and extracellular matrix (ECM) proteins [52]. Important cell types within the dermal layer include keratinizing cells, macrophages, fibroblasts, and adipocytes, which can communicate with each other in the skin environment [18]. The subcutaneous layer, which comprises adipocytes, MSCs, and connective tissue, is the last layer [53].

**Fig. 1:**
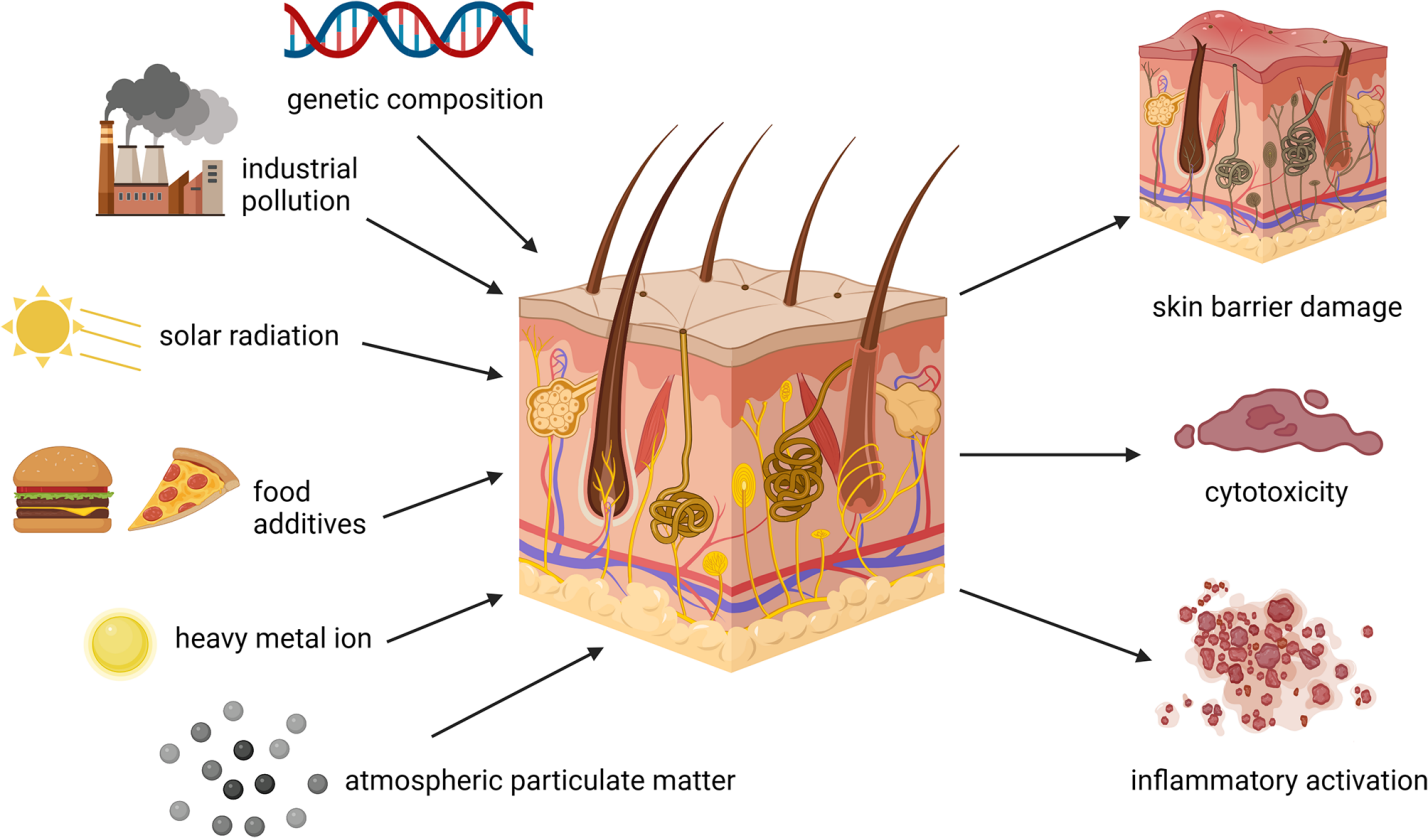
The effects of external factors on the skin. Skin cytotoxicity, skin barrier degradation, and inflammatory response activation can be caused by gene composition, environmental pollution, dietary additives, solar irradiance, heavy metal exposure, and particulate matter in the air

However, genetic composition, lifestyle, environmental pollution, food additives, solar irradiance, heavy metal exposure, and particulate matter in the air can cause skin cytotoxicity, weakening of the skin barrier, damage to matrix protein, and an active inflammatory response [54]. Loss of skin composition, as well as impairment of physiological functioning and natural structures, can result in skin abnormalities such as aging, hyperpigmentation, and poor skin healing after injury, all of which can have a detrimental effect on skin aesthetics [13] (Fig. 1).

### The physiological basis of skin wound healing

Skin wound repair is a complicated procedure that comprises many closely connected activities, which are broadly classified as inflammatory reactions, epithelialization, wound shrinkage, collagen deposition, and remodeling [55]. During the inflammatory response stage, immune cells prepare the wound for healing by eliminating pathogens, cell fragments, and apoptotic cells from the wound location [56]. Local monocytes travel into the wound, mature into macrophages, consume cellular debris and apoptotic cells, and produce a substantial amount of growth factors [57]. Inflammation increases the change of M1 macrophages into M2 macrophages. M2 macrophages enhance tissue repair and enormous ECM production by managing the multiplication and migration of keratin-forming cells, fibroblasts, and endothelial cells [58, 59]. Afterwards, the earlier formed wound matrix will gradually be replaced by granulation tissue, which contains capillaries, fibroblasts, and collagen bundles and serves as a scaffold for cell migration and growth [60]. Then, entering the epithelialization stage, keratinocytes migrate to the damaged dermis and reestablish the epithelial barrier function [61]. Cells rapidly proliferate, and new vessels and epithelium emerge. Afterwards, fibroblasts differentiate into myofibroblasts and contract the wound. During the collagen deposition stage, high concentrations of immature type III collagen are first released by fibroblasts into the stroma [62]. During the final remodeling phase, fibroblasts continue to secrete collagen. Over time, fibroblasts release matrix metalloproteinase (MMP) to remodel type III collagen into type I collagen, allowing the wound to seal. Collagen fibers gradually arrange, and when the wound’s tensile strength rises, the wound’s healing is complete [43, 63]. Throughout the process, many skin cells, like fibroblasts, adipocytes, endothelial cells, keratinocytes, macrophages, and other immune cells, interact to promote wound healing [64]. Among them, proliferation, migration, differentiation, and apoptosis of epidermal keratin-forming cells and dermal fibroblasts with damaged healing functions are the major causes [65].

Chronic wounds are defined as those that are deep, full, or partial thickness injuries and fail to recover within six weeks. They heal slowly and are linked with severe fibrosis, which can result in hyperplastic scars and keloids in some people [66]. Aside from its bad visual appearance, the tissue near the scar lacks several fundamental dermal components, like glandula sebacea, folliculus pili, and sensory nerve receptors [67]. There are several risk factors for the formation and maturation of scarring, including excessive collagen deposition, reduced fibroblast apoptosis, delayed keratinocyte function, increased transforming growth factor β1 (TGF-β1) expression, excessive angiogenesis, prolonged inflammation, and even aging [68]. Early management of the inflammatory reaction is crucial for renewal since unresolved long-term inflammation favors scar formation over regeneration [69–71]. Keloids and proliferative keloids are fibrous, proliferative malignant processes caused by excessive collagen and ECM protein buildup [72–74]. Through exosome-mediated intercellular communication, M2 macrophages are essential for the creation of permanent scars [18].

### The physiological basis of skin aging

The skin ineluctably loses structural and functional features due to a variety of internal and external factors. External factors, like airborne pollutants, lifestyle decisions, and notably UV radiation, are the principal causes of skin aging [75]. Aging reduces skin elasticity and changes skin thickness and collagen tissue, leading to wrinkles [76]. UV-induced photoaging is symbolized by sunburn, uneven pigmentation, roughness, dryness, and wrinkles, which are generated mostly by alterations in the ECM material [77–80].

Skin aging mechanisms are complicated, and they may include genetic mutations, DNA damage, cellular senescence, inflammation, and OS [75]. The accumulation of mutations in multicellular organisms may lead to age-related cell degeneration and death, resulting in the aging of the organism [81]. Age-related deficiencies in stem cells’ DNA repair machinery can result in chromosomal rearrangements or mutations that impair epidermal stem cells’ capacity to self-renew and thus accelerate the aging of the skin and/or the development of cancer [82]. OS has been demonstrated to have a major impact on the aging of the skin, and antioxidants like melatonin, vitamin C, and glutathione have the potential to aid skin renewal [75]. Excess reactive oxygen species (ROS) can directly harm cell function and structure, regulate inflammatory reactions, damage genetic components, and speed up the aging process of the skin [83]. MMPs are important regulatory targets of ROS-induced skin aging because they regulate the breakdown of numerous ECM components, especially collagen [84]. Human dermal fibroblasts (HDFs) are cells that primarily synthesize structural elements like pre-collagen and elastic fibers [85]. Aging alters the amount and growth of HDFs, decreases collagen production and repair, and speeds up MMP destruction of the existing skin matrix [18, 86]. ROS-stimulated MMP synthesis is mediated by the mitogen-activated protein kinase (MAPK) signaling cascade, which includes p38, extracellular signal-regulated kinase, and c-Junn-terminal kinase. Then the transcriptional factor activator protein 1 (AP-1) becomes activated and governs MMP-1, MMP-3, MMP-9, and MMP-12 transcription [87]. Another MMP-mediated signaling mechanism associated with the aging of skin is the TGF-β/SMAD system, which is hampered by TRII expression downregulation, resulting in decreased type I collagen formation [88]. Another transcription factor that is activated is nuclear factor-κB (NF-κB), which controls the response to photoaging and UV radiation by mediating the production of inflammation and MMP [89].

The interplay between melanocytes and keratin-forming cells in the epidermis is responsible for skin pigmentation. When exposed to UV radiation, keratin-forming cells release paracrine hormones such as endothelin-1 and α-melanocyte-stimulating hormone (α-MSH), which stimulate melanocytes to produce melanin [90]. Appropriate melanin serves as a natural sunblock, but excess melanin production, on the other hand, can lead to hyperpigmentation, which presents as UV-related pigmentation disorders such as solar freckle disease and melasma [91, 92]. When excessively exposed to UV light, fibroblasts age and create a number of skin aging-associated secretory proteins, including differentially expressed secretory factors that control melanogenesis. UV-irradiated fibroblasts, in particular, generate stem cell factor and secrete frizzled-related protein-2, which alter melanogenesis and contribute to the hyperpigmentation seen in solar freckles or melasma [93, 94].

## Mesenchymal stem cells and their secretory group

### Mesenchymal stem cells

A major challenge in the field of healthcare is the damage to tissue that results from illness, aging, trauma, and other causes. Regenerative medicine seeks to solve this problem by regenerating injured tissues [95, 96]. Stem cells are crucial to many regeneration procedures because they are able to differentiate into specific kinds of cells [97]. They are theoretically able to infinitely renew themselves under appropriate conditions and can maintain, produce, or restore injured tissue, which is difficult for other treatment methods to achieve [98, 99]. Compared with ESCs and iPSCs, MSCs have no ethical issues and possess stable cell phenotypes and a low immune status, which can reduce tumor risk and improve survival rate. Therefore, MSCs’ clinical applications are safer [28, 100].

**Fig. 2:**
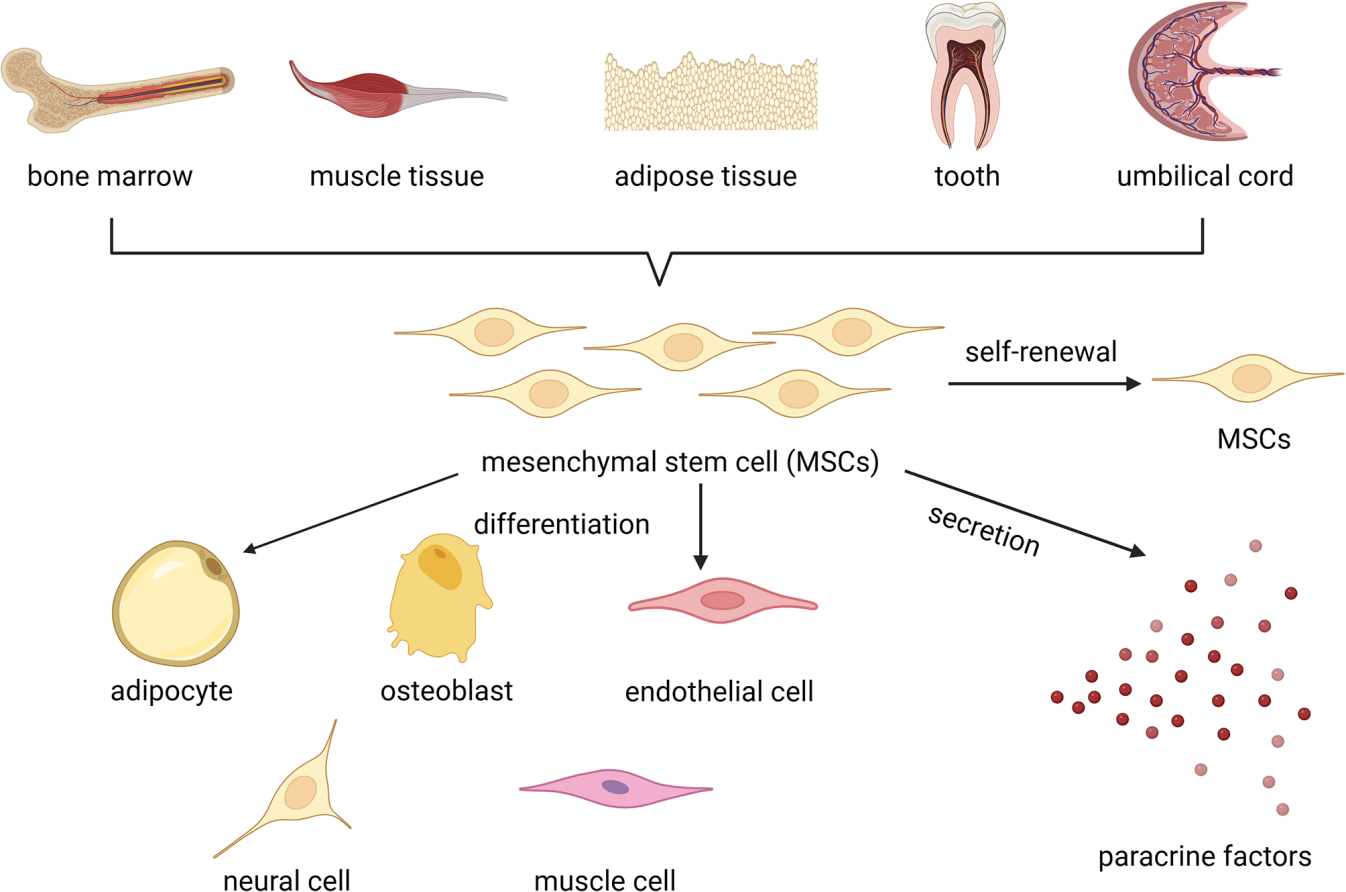
The source and functioning mechanism of MSCs. MSCs can be derived from a variety of tissues, like bone marrow, muscle tissue, adipose tissue, teeth, and umbilical cord, etc. MSCs can function through self-renewal, differentiation, and secretion

MSCs are pluripotent stem cells, they come from a range of body tissues, including bone marrow, umbilical cord, muscle, adipose tissue, and teeth [101, 102]. MSCs from various tissues have distinct biological characteristics, as evidenced by differences in differentiation ability and secreted factors (Fig. 2). MSCs may be used to treat soft tissue filling and revitalization, hair regeneration, scar reduction, and skin anti-aging. Some research has found that MSCs are able to enhance skin health by increasing skin thickness, collagen formation, and minimizing wrinkles [76]. Mechanically, MSCs may be found at the site of damage and release wound repair cytokines like platelet-derived growth factor (PDGF), insulin-like growth factor 1 (IGF-1), and interleukin-8 (IL-8), controlling inflammatory cells and decreasing fibrosis [103, 104]. In addition, it can regulate the immune reaction and stimulate tissue regeneration by secreting growth factors, chemokines, cytokines, and angiogenic factors [105]. Adipose-derived stem cells (ADSCs) and bone marrow mesenchymal stem cells (BMMSCs) have been investigated and used to limit scar formation, stimulate collagen production, enhance skin tone, and fight aging [106, 107]. According to the single cell map, ADSCs have less heterogeneity and rely less on mitochondrial metabolism for energy production than BMMSCs, resulting in improved stem cell maintenance and resistance to apoptosis [108]. ADSCs can be employed alone or in conjunction with interstitial vascular fraction to treat skin damage repair in vitro, such as lowering wrinkles, facial scars, antioxidant activity, and blocking melanin formation, leading to skin whitening [109–115]. Combined with other techniques, including carbon dioxide laser surface repair and cultured fibroblasts, ADSCs have shown skin-rejuvenating effects [116]. In comparison to ADSCs, the production of BMMSCs is more intrusive and damaging to patients. However, BMMSCs are more capable of self-renewal, differentiation, and immunological control [76]. For chronic wounds, BMMSCs move to the wound site between 7 and 8 weeks or 16–20 weeks after intravenous treatment, boosting pro-collagen production [117, 118]. Amniotic fluid stem cells and umbilical cord-derived stem cells (UMSCs) are two more sources of stem cells [119]. They are highly successful in restoring skin and immunological compatibility. It cannot, however, get an adequate amount of cells for therapy [120].

There are numerous treatments for improving skin health and treating skin problems, such as skin care, laser therapy, medicine, radiation therapy, and surgery [17]. However, because of the complicated nature of skin disorders, which involve many cell types and growth factors, these therapeutic mechanisms are relatively simple and have not achieved the desired skin repair effect [18]. MSCs not only regenerate tissue and restore damaged skin, but they also have various functions such as regulating immunity, reducing inflammatory reactions, and promoting angiogenesis. They have diverse treatment mechanisms, minimal trauma, significant preclinical effects, and no obvious toxic side effects in current research. Therefore, they can be used as a reliable alternative therapy [95, 121].

### MSCs secretory group

MSCs can secrete or shed numerous growth and trophic substances into the extracellular environment, creating the so-called secretome. This includes the soluble fractions and the extracellular vesicle (EV) fractions. EVs are important in the delivery of different genetic materials and proteins [122–126].

**Fig. 3:**
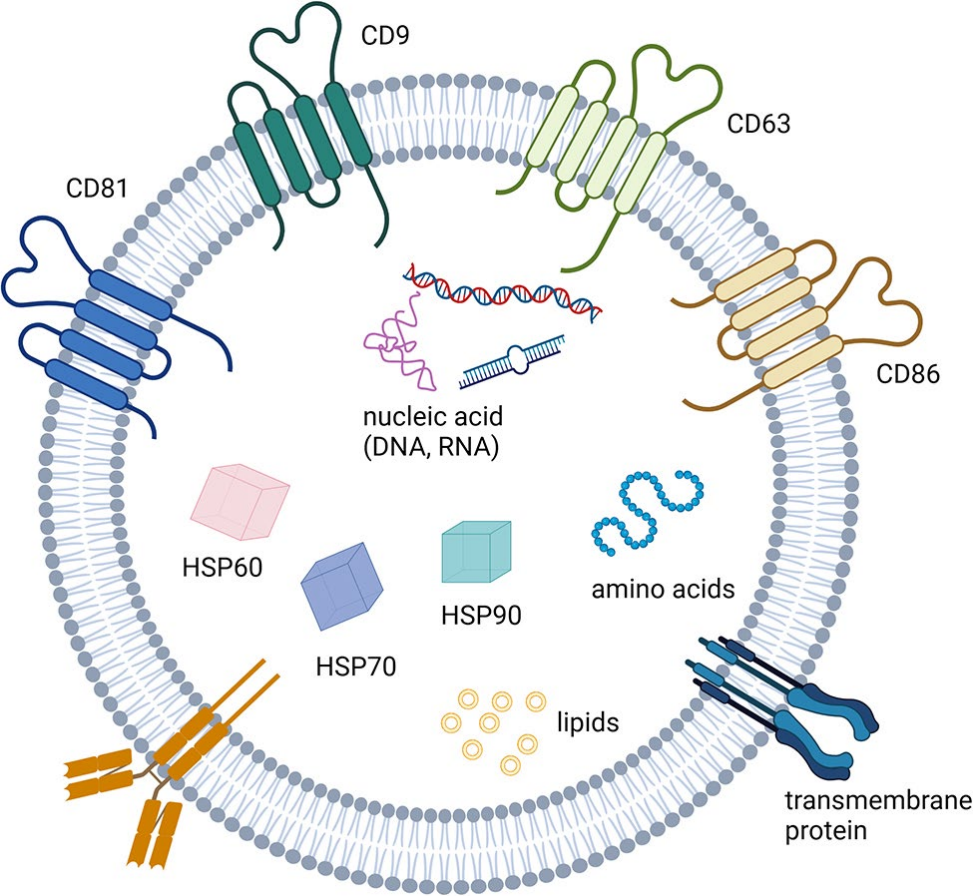
Hallmarks of exomes. Exo membranes contain tetraester proteins (CD9, CD63, CD81, and CD86), and transmembrane proteins. Exo contains heat shock proteins (HSP60, HSP70, and HSP90), nucleic acids, amino acids, and lipids

**Fig. 4:**
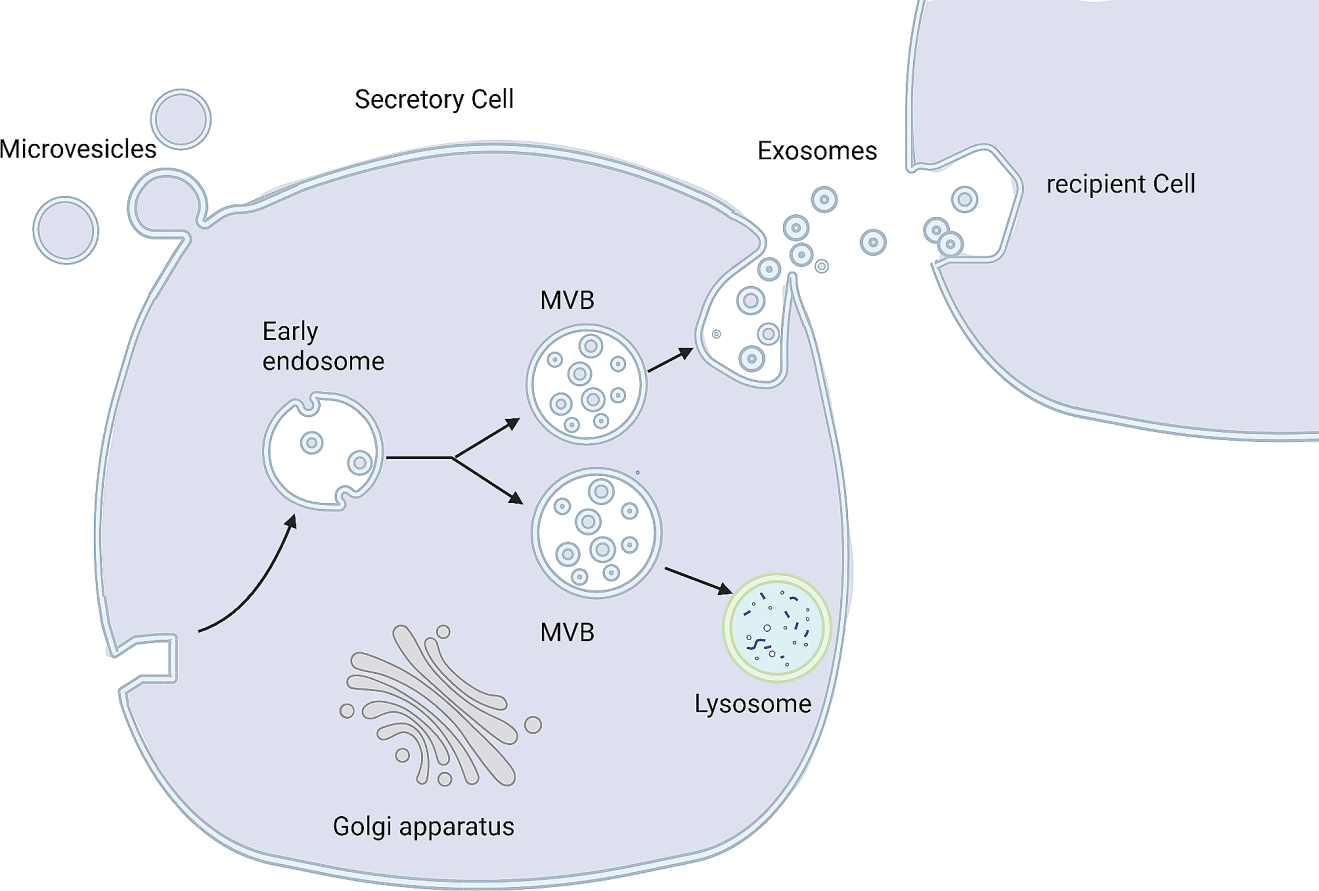
Formation and secretion of exosomes. The secretory cells are stimulated to transform early endosomes into multivesicular bodies, which secrete exosomes via fusing with cell membranes, and then exosomes carry various substances into recipient cells

EVs are divided into exosomes (Exo), microvesicles (MV), and apoptotic vesicles based on their size, content, and origin [104]. Among them, Exos have been extensively studied, which are tiny particles (40–120 nm in size) formed by multivesicular bodies (MVB). Exos include a variety of physiologically active macromolecules, including nucleic acids (such as miRNA, IncRNA, CircRNA, and DNA), proteins, and lipids that are important in cellular bioregulation [122, 127–129]. Tetraspanins (CD9, CD63, CD81, and CD86), membrane-linked proteins, and heat shock proteins (HSP60, HSP70, and HSP90) are abundant in Exos [127] (Fig. 3). Exos act by injecting their contents straight into cells, avoiding the requirement for specialized receptor expression [122, 128]. Exos may therefore serve as intercellular communication carriers, helping to overcome biological boundaries [130]. Exo possesses unique proteins and nucleic acids, depending on the origin of the cell, that support tissue regeneration through intercellular communication and are engaged in the control of apoptosis through immunomodulatory functions, anti-oxidative stress, and other mechanisms [127, 128] (Fig. 4).

Exo could modulate vital biological functions such as cell division, migration, differentiation, and death [18]. Studies have shown that endogenous exosomes shuttle through many types of skin cells and that their mediated messaging and intercellular contact are required for maintaining cell function and tissue homeostasis [131]. Exo from stem cells is expected to be a useful treatment in regenerative and cosmetic medicine, particularly in scar avoidance and reduction, pigmentation modulation, and hair growth [18]. Unlike stem cells, exosomes are small, inactive substances that can be stored at -80 °C for over 6 months without toxic cryoprotectants while still functioning [130, 132]. There is no requirement to sustain cell viability and efficiency from production to storage to delivery [133]. They also may avoid problems associated with cell therapy, including the potential for poor cell survival, immunological rejection, age-associated genetic instability, functional inactivation, and unfavorable differentiation [134].

MSC lysates are cell breakdown products containing cell membrane surface proteins and cellular contents that have a direct effect on injured tissues; they are not immunorejected like cells and play a function in regeneration comparable to exosomes and cell supernatants [135]. MSC lysates have anti-apoptotic activity, reducing tissue damage and promoting regeneration by inhibiting apoptosis [136]. The lysate of dental pulp stem cells (DPSCs) is abundant in various cytokines that enhance the cellular development environment and encourage the production of collagen in the skin [137]. In terms of safety, no serious negative effects, such as allergic reactions leading to death, have been observed using MSC lysate [138]. However, the main limitation is that the active ingredients are not yet well defined.

## The effect and possible mechanism of MSCs in improving wound healing and scar repair

### MSCs promote wound healing

MSCs achieve the effect of promoting wound healing through various mechanisms. Firstly, what works is the multi-directional differentiation ability of MSCs. MSCs may develop into a range of cells, for example, ADSCs can develop into adipocytes, endothelium cells, skeletal muscle cells, and smooth muscle cells to enhance skin wound healing [139, 140]. ADSCs are better suited to directed adipocyte growth than stem cells from the rest of the body. According to previous research on injury healing and regeneration, adipocytes can govern fibroblast recruitment and play a key role in skin reconstruction [141]. Previous studies have shown that fibroblasts are reduced in animals with fat accumulation problems, and adipocytes may indirectly encourage fibroblast recruitment by regulating the generation of unknown fibroblast precursor cells in the skin. There is also evidence that direct intercellular communication between adipocytes and fibroblasts may influence fibroblast migration during skin wound repair [141]. Dermal adipocytes are critical in the initial stages of injury-induced immune activation. Shook et al. discovered that adipocytes at the wound site dilate and then shrink due to adipose triacylglyceride lipase dependent lipolysis. The products of lipolysis recruit immune cells, which are necessary for effective wound closure [142]. Adipocytes have also been shown to recruit fibroblasts necessary for wound healing and promote ECM deposition [143]. After the wound heals, the adipocytes along the wound’s edge undergo lipolysis, which releases free fatty acids, activates macrophages, induces angiogenesis, and promotes tissue repair [144]. ADSCs can grow into vascular endothelial cells. Under the stimulation of bone morphogenetic protein 4 (BMP-4) and TGF, ADSCs can develop into smooth muscle cells (SMCs) [145, 146]. Because smooth muscle is essential for blood vessel physiological performance, the creation of SMCs is necessary for the in vitro construction of blood vessels with correct physiological function [50, 147]. Blood vessels rely on SMCs for structural support and contraction [148]. The vascular system facilitates the transfer of nutrients and oxygen, and creates an inflammatory environment. Therefore, the formation of a new circulatory system throughout the regeneration and repair phase is critical for the entire healing process [149]. Damaged wound vascular reconstruction can impede healing and contribute to the development of chronic wounds [150]. ADSCs can also differentiate into skeletal muscle cells to promote tissue healing [151].

The paracrine function of MSCs is also crucial. MSCs can colonize at the site of injury and express high levels of wound-healing cytokines like IGF-1, PDGF, and IL-8, thereby regulating inflammatory cells and down-regulating fibrosis [103, 104]. ADSCs, in particular, may produce almost all of the growth factors required for healthy wound repair, like vascular endothelial growth factor (VEGF), hepatocyte growth factor (HGF), basic fibroblast growth factor (bFGF), and PDGF. They can also stimulate the excretion of those growth factors in chronic wounds in a hypoxic environment [50]. MSCs also promote new blood formation, modulate the immune response, and inhibit excessive inflammation. Without neovascularization, acute injuries could turn chronic, and EVs generated from different MSC sources have been demonstrated to induce angiogenic responses in vivo [152–155]. MSCs enhance angiogenesis and facilitate the growth of a functioning vascular system during this stage of wound healing [156–158]. MSCs may promote neovascularization in adults by releasing pro-angiogenic factors like VEGF, hypoxia inducible factor-1 (HIF-1), epithelial growth factor (EGF), and C-X-C motif chemokine ligand 12 (CXCL12) [159], and secreting various molecules that improve vascular stability and protection [160, 161]. ADSCs release angiogenic cytokines such as TGF-β, VEGF, HGF, bFGF, PDGF, and angiopoietin-1 (Ang-1), which increase angiogenesis in granulation tissue, enhance local blood circulation, accelerate tissue regeneration at the ischemic site, and shorten healing time [162]. The continuation of an inflammatory reaction that should have halted after the inflammatory phase is one reason for wound healing problems, resulting in a delayed healing process [50]. ADSCs diminish pro-inflammatory factors like tumor necrosis factor-α (TNF-α) and interferon-γ (IFN-γ) while increasing anti-inflammatory ones like interleukin-4 (IL-4) and interleukin-10 (IL-10) [163]. Systematically infused BMMSCs migrate to local wound sites, interact with the inflammatory microenvironment, and induce macrophage polarization toward the M2 phenotype [164, 165]. ADSCs regulate cytokines by suppressing T-lymphocyte activation and B-lymphocyte apoptosis [166]. ADSCs can also suppress the immune response via direct cell-to-cell contacts and paracrine cytokines such as IL-10, HGF, indoleamine 2,3-dioxygenase 1, and TGF-β [50]. MSC therapy improves fibroblast survival and migration as well as fibroblast ECM deposition, which improves healing [167, 168].

**Fig. 5:**
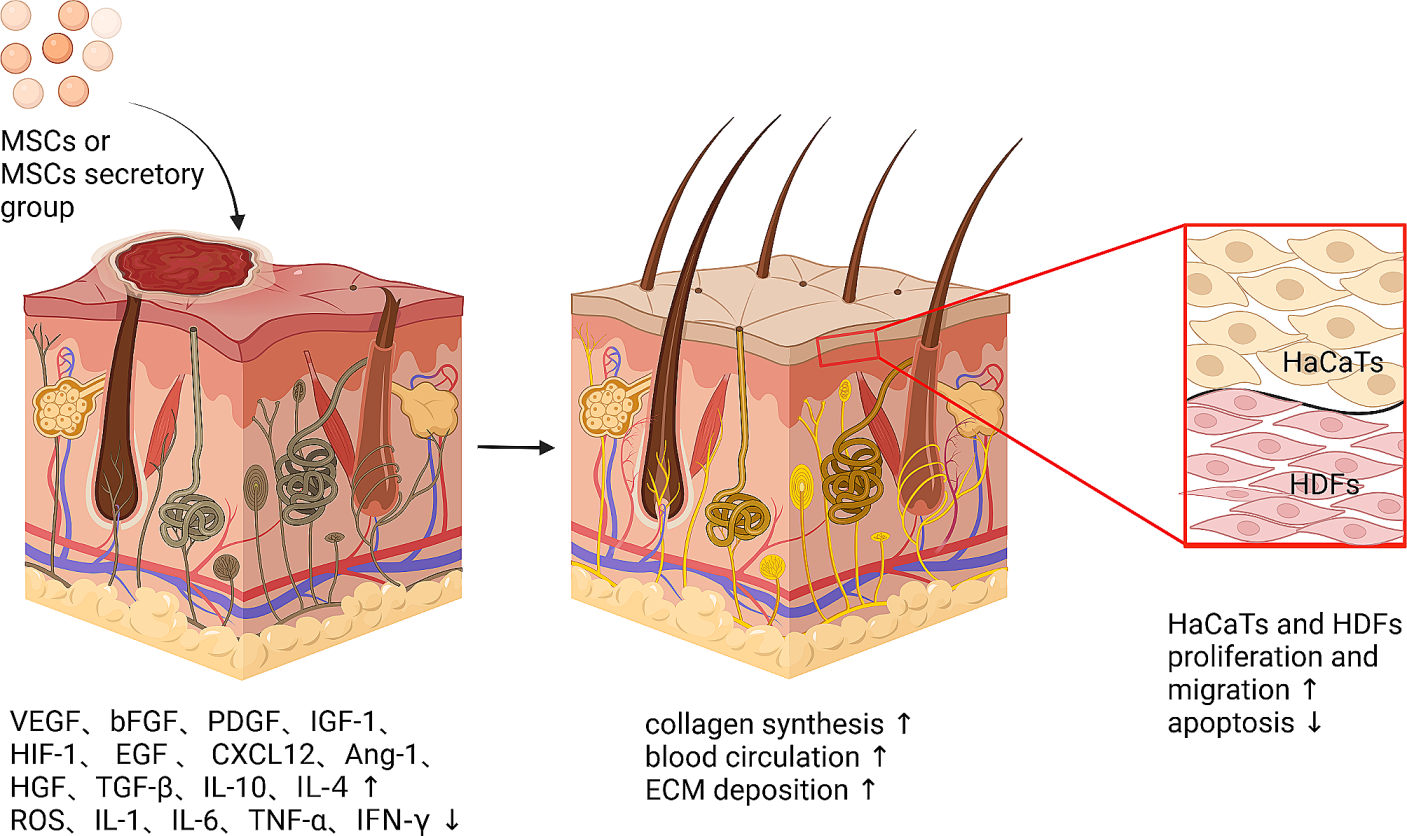
The mechanism by which MSCs promote skin wound healing. In damaged skin, MSCs promote the secretion of VEGF, bFGF, PDGF, IGF-1, HIF-1, EGF, CXCL12, Ang-1, HGF, TGF-β, IL-10, IL-4, inhibit the secretion of TNF-α, IFN-γ, IL-1, IL-6, and the level of ROS, resulting in enhanced differentiation migration and reduced apoptosis of HaCaTs and HDFs, thus promoting skin collagen synthesis, hematopoietic formation, and ECM deposition, leading to the tendency of skin wound healing

Due to the fact that paracrine function is one of MSCs’ primary mechanisms of action, in-depth research has been conducted on the secretomics of MSCs. The following will provide a detailed introduction. For example, ADSC extracellular vesicles promote wound healing by increasing phosphorylation of aging biomarkers VEGF, VEGF receptor 2 (VEGFR2), and senescence marker protein 30 (SMP30) while inhibiting the creation of ROS and inflammatory cytokines like interleukin-1 (IL-1), TNF-α, and interleukin-6 (IL-6) [169]. ADSC-exos perform a crucial part in wound healing by acting on key target cells like HDFs and human immortalized keratinocytes (HaCaTs) through multiple signaling pathways [170]. Ma et al. treated HaCaTs with H_2_O_2_ to simulate skin damage and discovered that ADSC-exos can improve HaCaTs proliferation and migration, and prevent apoptosis via the Wnt/β-linked protein signaling pathway [171]. He et al. recently demonstrated that malat1-containing ADSC-exos promoted wound repair by stimulating the Wnt/β-linked protein pathway [172]. By upregulating the phosphoinositide 3-kinase/Akt (PI3K/Akt) pathway, ADSC-exos may promote and improve collagen production during skin wound healing [173]. Li et al. discovered that when diabetes rats were given exosomes from ADSCs with high expression of NF-E2-related factor 2 (Nrf2), the wound ulcer area of their feet was greatly reduced. Collagen formation is particularly vital in the initial phases of recovery, whereas matrix rebuilding is of greater significance later in the healing process [134]. ADSC-exos improves ECM remodeling and reduces scarring by modulating the ratio of type III/type I collagen, TGF-β3/TGF-β1, and MMP-3/tissue inhibitor of metalloproteinases 1 (TIMP-1) and promoting HDFs differentiation [174]. To prevent scar formation in an incision treatment model, ADSCs exosomes increase type I and III collagen formation early in the recovery phase while inhibiting collagen production later in wound healing [109](Fig. 5).

Bandages, hydrogels, and sponges are the main nanodrugs used to assist wound healing [175, 176]. Mozafari et al. designed thermosensitive hydrogel capsules to reduce the level of inflammation and promote wound recovery [177]. When BMMSCs were cultivated in hydrogels and administered to skin wounds in a mouse model, the therapy aided in wound healing, epithelial cell multiplication and re-epithelialization, and lowered inflammatory responses in serious skin lesions [178]. Graphene has good biocompatibility, which can stimulate cell proliferation and have antibacterial properties. Previous research has discovered that the interaction of graphene-based nanomaterials with cells involved in wound repair reactions might improve the selectivity of MSC Exos in regulating gene expression, thereby promoting wound healing [179]. Silver nanoparticle (AgNP)-based nanomaterials have been widely studied and have a wide range of applications. RPS-AgNPs nanocomposites synthesized by impregnating radiosterilized pig skin (RPS) with AgNPs suspension reduce bacterial growth and contribute to the survival and proliferation of MSCs [180]. The synthesis of AgNPs from the water extract of turmeric leaves and the biosynthesis of AgNPs through polycystis algae showed significant antibacterial and wound healing potential [181, 182]. CuS@BSA nanoparticles can induce MSCs to differentiate into fibroblasts, making them an effective tool for influencing MSC differentiation [183].

### MSCs promote scar repair

Plenty of research has proven that MSC can both improve wound healing and reduce scar formation. Fang et al. discovered that UMSCs decreased scar development and myofibroblast production in a mouse model of skin defects [184]. Liu et al. demonstrated that MSCs transplanted through the ear artery dramatically decreased proliferative scar development in a rabbit ear proliferative scarring model, implying that MSCs may have practical uses in regulating wound healing [185]. Similar to that, another experiment in a rabbit model found that local application of MSCs effectively reduced proliferative scar development by controlling inflammation [186]. In the rabbit model, Li et al. discovered that transplanting BMMSCs overexpressing TGF-β3 dramatically enhanced wound repair and decreased the production of skin scar [187].

MSCs paracrine activity is crucial in this regard. MSCs produce a variety of antifibrotic mediators and growth factors, including HGF, IL-10, and adrenal medulla [188, 189]. MSCs that migrate to the site of injury emit HGF and Prostaglandin E2, which inhibit myofibroblast differentiation and avoid epithelial-mesenchymal transition [190, 191]. MSCs may also influence the formation of ECM and fibroblasts for better scarring. Cecelia C. Yates et al. discovered that allogeneic MSCs transplantation increased fibroblast proliferation, migration, and ECM deposition, all of which are required for wound healing and reduced post-traumatic inflammation [167]. Similar to cutaneous tissue, MSCs signaling causes other nearby cells to form the right ECM [192].

**Fig. 6:**
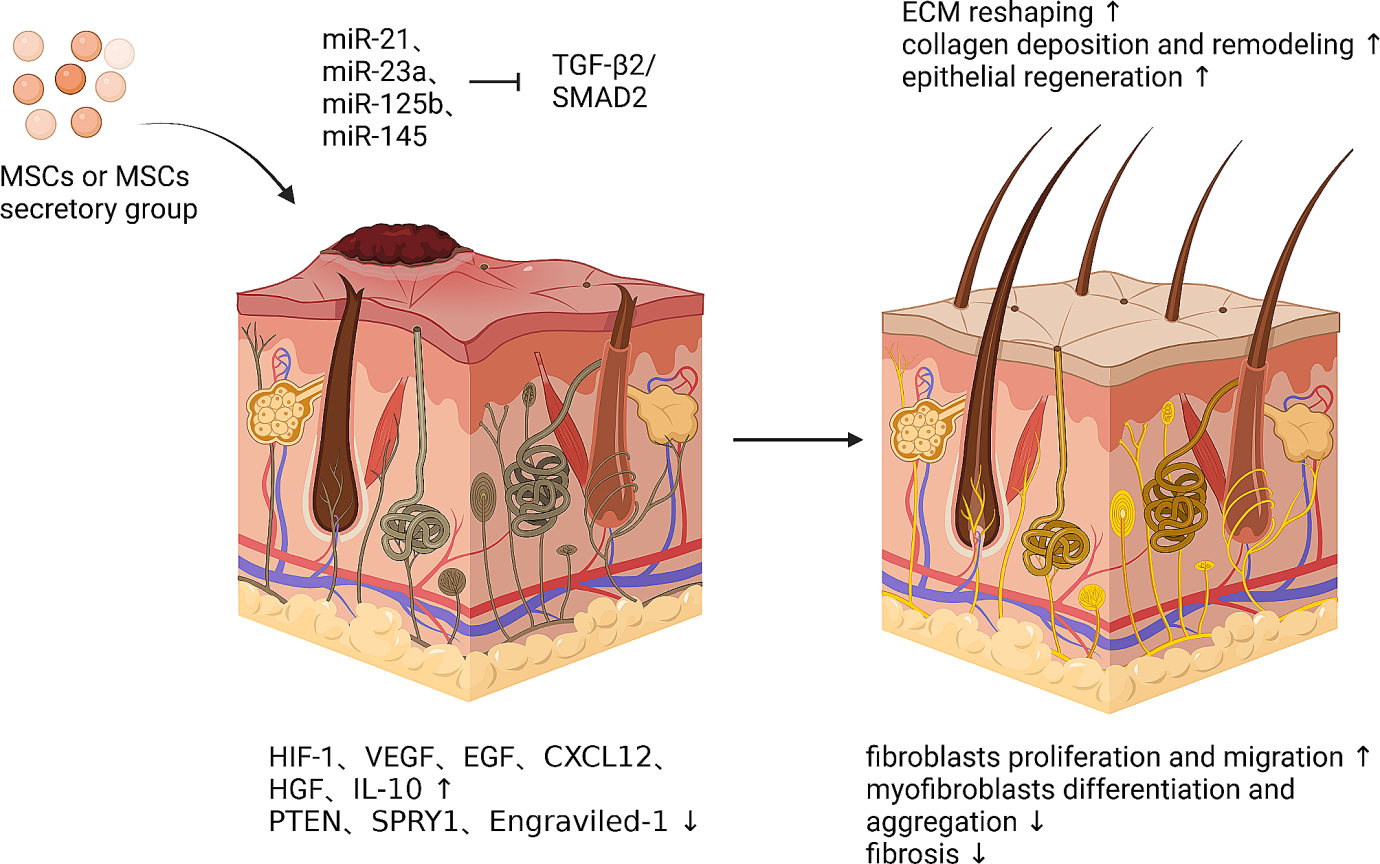
The mechanism by which MSCs promote skin scar repair. MSCs have the ability to stimulate the release of HIF-1, VEGF, EGF, CXCL12, HGF, and IL-10 in scarred skin while inhibiting the release of PTEN, SPRY1, and Engraviled-1. Exosomes secreted by MSCs contain miR-21, miR-23a, miR-125b, and miR-145 that can suppress the TGF-β2/SMAD2 pathway, promote fibroblast differentiation and migration, and inhibit myofibroblast differentiation and aggregation. This can reduce fibrosis and promote ECM remodeling, collagen deposition remodeling, and epithelial regeneration

MSC exosomes promote collagen deposition and have antifibrotic properties in proliferative scarring [193–195]. Wang et al. indicated in a mouse model that ADSC-exos improved ECM remodeling and scar-free healing. The underlying process may be connected to the modulation of the type III: type I collagen ratio, MMP3:TIMP-1, TGF-β3:TGF-β1, and the inhibition of myofibroblast differentiation [174]. Furthermore, in a mouse model with full-thickness skin injuries, ADSC-exos shortened healing time, promoted collagen synthesis, and reduced scarring by activating the signaling pathway of PI3K/Akt [173]. Hu et al. discovered that topical administration of human umbilical stalk plasma exosomes overexpressing miR-21-3p expedited re-epithelialization, decreased scar breadth, and improved angiogenesis in mouse skin wounds by reducing phosphatase and tensin homolog (PTEN), and sprouting homologue 1 (SPRY1) [196]. Zhang et al. revealed that placental MSC-exos-induced wound restoration may be done mostly by downregulating the Yes-associated protein signaling pathway, thereby inhibiting Engraviled-1 to reduce scar formation [197]. Yuan et al. discovered that exogenous miR-29a90-modified ADSC-exo treatment reduces scar growth by blocking the TGF-β2/SMAD3 signaling pathway [198]. Fang et al. revealed that UMSC-Exos enriched with particular microRNAs (miR-21, miR-23a, miR-125b, and miR-145) decrease myofibroblast production and anti-scarring by suppressing the TGF-β2/SMAD2 pathway [184]. In a mouse model of skin abnormalities, UMSCs-exos inhibits TGF-β2/SMAD2 pathway activity, decreasing myofibroblast differentiation and over-aggregation, and therefore reducing hyperfibrosis and scar formation [18]. These data suggest that MSC-exos, especially ADSC-exos, can modulate fibroblast activity, as well as collagen deposition or alignment, to promote scar-free patterns (Fig. 6).

In the application of nanomaterials, Zheng et al. found that MSCs-rich hydrogels helped skin wound healing and formed scar-free tissue with hair follicles [199]. When used in conjunction with a multifunctional polysaccharide-based dressing scaffold, ADSC-exos can accelerate recovery by increasing cell proliferation, granular tissue growth, collagen accumulation, re-epithelialization, and remodeling while decreasing scar tissue development and skin attachment regeneration [200]. Table 1 points out recent clinical research on the use of MSCs to treat different kinds of wounds. More clinical studies with MSCs transplantation are expected to be conducted in the future.

**Table 1:** Current clinical studies of skin wound healing and scar repair

Source	Research Contents	Finding	References
ADSCs	a phase I trial of seven participants with recurrent ulcers and ischemic limb injuries	ADSCs improve wound healing by lowering leg pain and ulcer size	Bura et al. 2014 [201]
ADSCs	treat chronic wounds with a biological bandage made of ADSCs and adult acellular collagen matrix	the dressing greatly increase dermal revascularization and wound reformation	Lafosse et al. 2015 [202]
UMSCs	a clinical trial with randomization and control to treat diabetic foot ulcers	three months after UMSCs implantation, ulcer is entire or progressing recovery	Qin et al. 2016 [203]
placental MSCs	local application of alginate sodium gel containing placental MSCs to ulcerative diabetes foot	the ulcer basically heals after three weeks	Zeng et al. 2017 [204]
ADSCs	treatment of diabetes foot with allogeneic ADSCs tablets based on hydrogel	the ADSCs group has a higher rate of full wound healing	Moon et al. 2019 [205]
UMSCs	the impact of serum-containing human UMSC medium on laser therapy recovery	reduce the recovery time after treatment for erythema and laser ablation treatment	Jihee Kim et al. 2020 [206]
ADSCs	the ability of ADSCs to accelerate the healing of skin wounds	ADSCs promote wound healing	Zhou et al. 2022 [207]
MSCs	utilize fibrin polymer sprays to apply cultured autologous MSCs to wounds	increase healing in those with chronic, non-healing lower limb wounds	Falanga et al. 2007 [117]
MSCs	give MSCs to wounds with matrix or hydrogel	accelerate healing and significantly improve clinical outcomes	Dash et al. 2009 [208]

## The role and possible mechanism of MSCs in promoting skin rejuvenation

By increasing fibroblast growth and biological activity, lowering inflammation and ROS, boosting collagen production, and decreasing MMP expression, MSCs have also demonstrated promising results in the therapy of aging skin [75]. ADSCs can be employed individually or in conjunction with stromal vascular fraction to repair skin defects such as face scars, antioxidants, wrinkles, and melanin synthesis, resulting in skin whitening [110–115, 209]. Nuclear receptor-interacting protein 1 (Nrip1), according to Hu et al., plays an important function in aging. Treated with ADSCs, skin aging was slowed with decreased expression of inflammation-related genes (IL-6, p65, and IL-1α), aging-related genes (p21 and p53), and growth factor-related genes (Igf1, mTOR) under Nrip1 knockdown [210]. ADSCs have exhibited anti-aging and skin-rejuvenating characteristics when combined with other approaches like CO2 laser surface repair and cultivated fibroblasts. Potential connections include the MAPK and TGF-β pathways, which modulate MMP production and ECM formation [75, 76]. ADSC-conditioned medium (ADSC-CM) was discovered to reduce ROS production and suppress photoaging through inhibiting IL-6 and MMP-1 production and enhancing the antioxidant gene heme oxygenase-1 (HO-1) expression [211]. Hwang et al. discovered NF-κB pathway activation in another investigation. Both ROS production and MMP expression were improved in the therapy group using neural stem cell-conditioned medium (NSC-CM) and its released components, TIMP-1 and TIMP-2 [212]. The activation of the DNA repair enzyme Rad50 and consequent suppression of the DNA damage biomarker γ-H2AX serve to highlight the protective impact of NSC-CM [212]. In the treated skin tissue, higher levels of tissue proteinase K and MMP-12, as well as enhanced M2 macrophage infiltration were found, suggesting elastinolytic and perhaps anti-inflammatory effects [213].

In vitro tests have revealed that HDFs are shielded from oxidative damage by ADSC-CM [214]. In an in vitro study of UVB irradiation, Li et al. found that ADSC-CM effectively upregulated the production of antioxidant response factors, like TGF and HO-1, while downregulating the activity and transcription of UVB-induced signaling pathways, like AP-1, MAPKs, and NF-κB [211]. Therefore, ADSC-CM exerts protective properties on HDFs and HaCaTs against UVB-induced photoaging [134]. Guo et al. reported that platelet-derived growth factor AA (PDGF-AA), which is present in ADSC-CM, also activates the PI3K/Akt signaling pathway, mediating ECM deposition, photoaging-induced proliferation, and HDFs remodeling [215]. The results suggest that well-prepared ADSC-CM has a positive preventive effect on preventing intrinsic and extrinsic aging damage of HDFs to some extent. Also, the results clarify that PDGF-AA may help to obtain better results with other elements of ADSC-CM [134].

Nevertheless, the components of ADSC-CM are quite complicated and do not work synergistically to achieve anti-aging goals. Exosomes are important components of ADSC-CM and may have positive independent or synergistic effects [134]. Exosomes have the ability to facilitate intercellular communication as well as control HDFs characteristics [18]. Exosomes formed by three-dimensional growth of HDFs spheres (3D-HDF-exos) boost type I procollagen expression while decreasing MMP-1 expression via TNF-α downregulation and TGF-β upregulation [216]. Exosomes transport a variety of membrane proteins and cytoplasmic components, and they modulate pigmentation in both healthy and pathological situations through controlling gene expression and enzyme activity [18]. 3D-HDF-exos led to greater amounts of skin collagen deposition in vitro and in a naked mouse photoaging model than BMMSC-derived exosomes. Thus, 3D-HDF-exos may control cutaneous fibroblasts, stimulate appropriate collagen formation, reduce inflammatory responses, and have anti-aging properties [217]. Oh et al. reported that in UVB-driven photoaging and normal aging models, human iPSC-exos ameliorated genetic and phenotypic abnormalities in photoaging HDFs. The favorable benefits of iPSC-exo were accomplished mechanistically by lowering MMP1/3 and senescence-associated β galactosidase expression while increasing type I collagen production in aged HDFs [218]. Wang et al. discovered that ADSCs and their CM effectively reduced UVB or α-MSH-induced hyperpigmentation in B16F10 cells in mice ears or human skin substitutes in vivo and in vitro by suppressing melanin formation and boosting melanosome breakdown. They also found that miR-199a and miR-181a-5p extracted from ADSCs exosomes significantly suppressed melanogenesis via inhibiting microphthalmia-associated transcription factor, a major regulator that controls melanogenesis and promotes melanosome degradation through activation of autophagy [219].

**Fig. 7:**
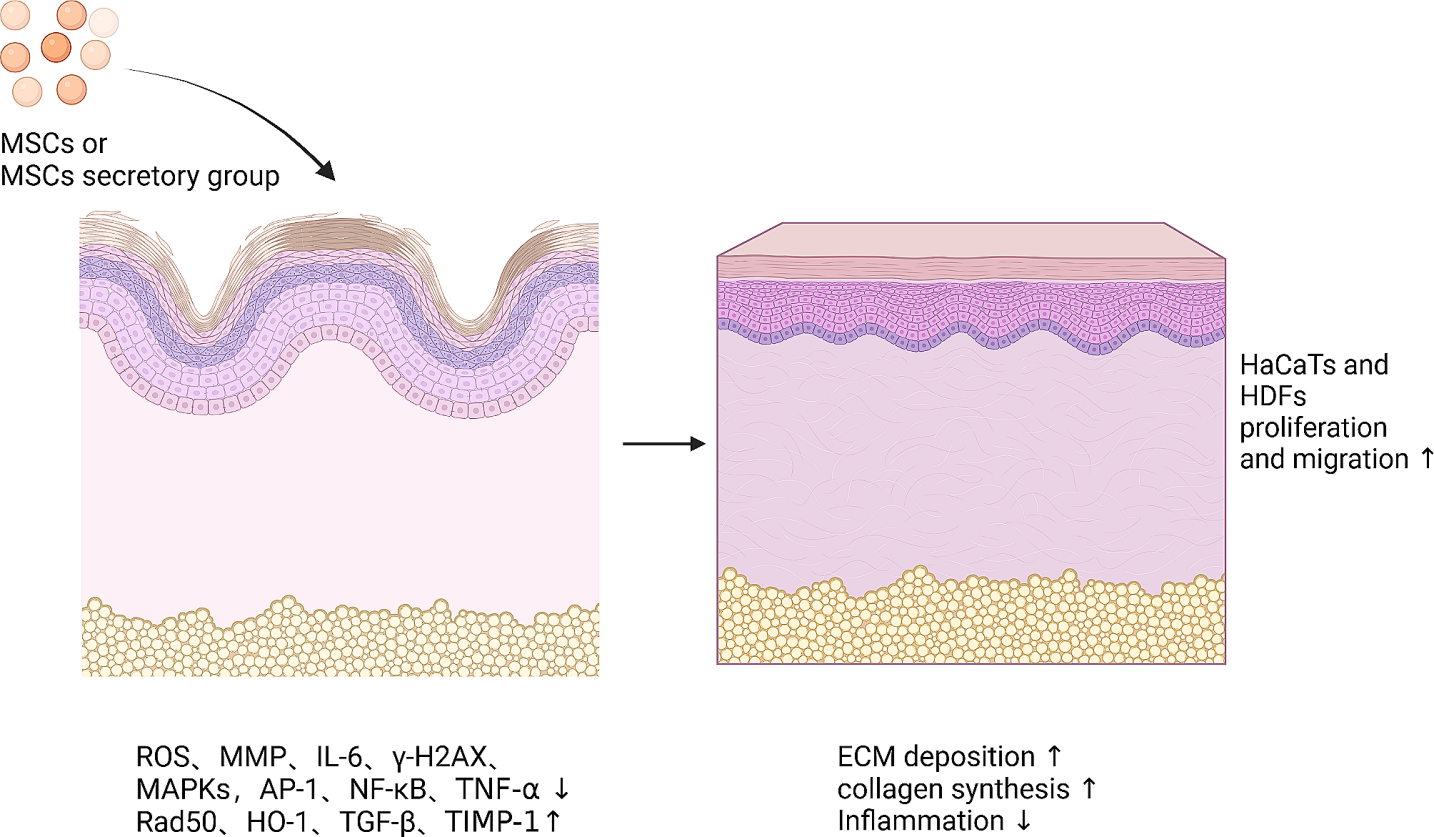
The mechanism by which MSCs promote skin rejuvenation. In aging skin, MSCs stimulate the release of Rad50, HO-1, TGF-β, and TIMP-1, lower ROS levels, suppress inflammatory responses, inhibit the expression of MMP, IL-6, γ-H2AX, MAPKs, AP-1, NF-κB, and TNF-α, and promote the differentiation and migration of HaCaTs and HDFs, which in turn stimulate the synthesis of collagen and ECM, leading to skin rejuvenation

Bae et al. discovered that exosomes expressing mmu-miR-291a-3p mechanically corrected HDFs aging via the TGF-β receptor 2 pathway. According to this research, ESC-exo mmu-miR-291a-3p has the ability to slow the aging of the skin [220]. Some research has indicated that exosome has numerous growth factors linked to skin regeneration, like EGF and bFGF [221]. According to the activation of Col-1 and glutathione peroxidase-1 and a decrease of MMP-1, hucMSC-derived extracellular vesicles prevent photoaging via lowering ROS production, increasing fibroblast growth, and avoiding the arrest of the cell cycle [222]. Using ADSC-exo treatment, Liang et al. published PCR data indicating enhanced type I collagen mRNA expression and reduced MMP-1, MMP-3, and type III collagen expression [223]. Meanwhile, TGF-β1 and TIMP-1 expression were upregulated, leading to the restoration of photodamaged dermal fibroblasts [224] (Fig. 7).

Liposomes, vesicles, solid lipid nanoparticles, and metal nanoparticles are some of the most popular nanocarriers utilized in cosmetics [225, 226]. The active ingredients are often packaged in nanocarriers to promote skin absorption and achieve better cosmetic and therapeutic effects [227]. Nanomaterials have been employed in sunscreen for their excellent encapsulation capabilities, better stability of encapsulated bioactive ingredients, and controlled release. Nanoparticles based on zein exhibit antioxidant effects on matrix metalloproteinase MMP-1 [228]. Natural compounds and poly ε-caprolactone nanofibers can combat stem cell aging and prevent aging caused by ultraviolet radiation [229]. Alginate/gelatin hydrogel bioink and glucosamine-based supramolecular nanotubes can maintain the pluripotency of MSCs [230, 231]. Gold nanoparticles can be used as anti-aging components [232]. Nanoparticles created from Rosa Floribunda Charisma could be an organic source of a new anti-aging ingredient for the skincare and cosmetics industries [233]. Future research can consider the combination therapy of multiple nanomaterials and MSCs to achieve higher anti-aging effects. Table 2 presents recent clinical studies using MSCs therapy to promote skin rejuvenation. More related research is expected to be conducted in the future.

**Table 2:** Current clinical studies of skin anti-aging/rejuvenation

Source	Research Contents	Finding	References
amniotic MSC**s**	the effects of amniotic MSC-CM on photoaging	significant improvement in photoaging after treatment	Prakoeswa et al. 2019 [234]
amniotic fluid MSCs	use stem cell conditioned media together with acupuncture for facial rejuvenation	enhance skin texture, improve collagen and elastin production, and help regulate face aging	El-Domyati, M. et al. 2020 [235]
ADSCs	use ADSCs for ECM regeneration in patients with solar elastosis	obtain the full recovery of solar elastosis	Luiz Charles-de-Sá et al. 2020 [213]
ADSCs	investigate the effect of protein isolates from ADSC medium applied via microneedles to Asian skin	present rejuvenating and whitening efficacy	Wang et al. 2018 [236]
red deer umbilical cord lining MSCs	compare the efficiency of red deer MSCs extract to a vehicle for face rejuvenation	effective restorative function on aging faces	Marwan Alhaddad et al. 2019 [237]
ADSCs	analyze the impact of combining niacinamide with ADSC-CM in topical post-laser treatment	valid anti-aging impact	Lee et al. 2020 [238]
ADSCs	nano method implantation of adipose tissue containing stem cells for skin therapy in patients	better skin quality, reduced spots and pigmentation	Omeed Memar et al. 2014 [239]
MSCs	bioactive substances made from MSC-CM to address dark under-eye circles	reduce the under-eye dark circles and improve the overall look of the eye zone	Samatha Bhat et al. 2022 [240]

## Conclusions

Cutaneous medical aesthetics has gained more attention recently, particularly in relation to skin renewal and scarless skin wound healing. Mesenchymal stem cell therapy has demonstrated significant promise in encouraging skin repair and rejuvenation via paracrine actions, immunological regulation, inflammatory management, and tissue differentiation. The paracrine influence of MSCs is the most important, controlling intercellular contacts via cytokines such as VEGF, bFGF, and PDGF, and extracellular vesicles such as exosomes, of which exosomes have been the most extensively studied. The newly proposed stem cell lysates have also shown promising therapeutic effects, helping to combat skin photoaging and improve skin condition. Unfortunately, there isn’t much research being done on this topic right now.

Clinical application of MSC therapy is still far off, despite the fact that in vitro and in vivo trials have shown great promise. First of all, basic cell and animal research cannot correctly reflect human situations. This is due to species variances in human and animal skin tissues, resulting in differences in dermatological and healing mechanisms between human and animals. For instance, mouse skin is laxer than human skin, and mice recover via wound contraction, which differs markedly from human wound healing [241]. Another issue is the time it takes for scar onset. Most mouse models produce mature hyperplastic or keloid scars weeks to months after incisional injuries [242]. Excessive scarring may occur after a few months in people, with biomolecular proof of disease progression after one year; keloids and hyperplastic scars have been found to return months to years after successful treatment [243–245]. Thus, the brief life of the mouse model could not provide enough evidence to determine whether the positive impacts of MSCs treatment persist. Future investigations of porcine models with extensive follow-up periods may aid in this [246]. Furthermore, differences in clinical trials like cell source, dosage, and drug delivery technique make direct comparisons between studies difficult. From differentiation potential to immunomodulatory capacity, MSCs of different tissue origins differ greatly in their biological properties [247–249]. Future research is needed to determine which cell types have the most effective therapeutic effects. This data will be valuable for MSCs quality control in clinical settings, ensuring predictable repair results [250]. It is also critical to create appropriate and consistent patient selection criteria, which will serve as the basis for subsequent treatment comparisons. Furthermore, the number of patients currently participating in completed and ongoing clinical trials is minimal, most clinical trials lack adequate controls, and no conventional treatment is utilized as a positive control to establish the efficacy of a beneficial MSCs-based treatment. Thus, future high-quality clinical studies, particularly massive, randomly assigned, double-blind, controlled clinical studies with a lengthy follow-up period, are urgently needed [250]. Last but not least, the safety of stem cell treatment is still being researched. The use of MSCs involves some risk, which can’t be overlooked while developing clinical protocols. Genomic instability has been reported to accumulate in the progeny of MSCs during ex vivo amplification [26, 27]. As a result, adequate cell passages ought to be found while performing clinical studies; in particular, graft cell genotype should be assessed prior to cell transplantation. MSCs have been shown in animal models to migrate to tumors and promote tumor development and progression [251, 252]. Although current clinical studies have not reported any occurrences of tumor formation following in vivo MSCs delivery to our knowledge, it is still vital to exclude any unfavorable effects through cell monitoring and long-term follow-up.

Therefore, considering that the primary wound repair mechanism for MSCs-based treatments is paracrine impact and that cell-free therapy is safer, MSC- conditioned medium is regarded as a potential technique to aid chronic wound repair since it contains several paracrine substances released by MSCs during in vitro culture, as demonstrated by several animal tests [253–256]. The exosomes isolated from MSC-conditioned media are the hot spot of stem cell therapy, which reduces the possibility of inadequate differentiation or cancerous transformation of transplanted cells, making it a safer technique [257]. MSCs lysate also have the same advantages, making them safer and more convenient for storage and transportation. As a result, future studies can focus on MSC exosomes and lysates. More preclinical and clinical researches are needed in the future, especially for clinical assessments of both the safety and effectiveness of MSC conditioned media and lysates [250].

To summarize, MSCs therapy offers a very broad applicability promise in dermatology and cosmetic medicine, but the particular mechanism of action remains unknown, and high-quality clinical trials are uncommon. MSCs have varying biological features based on their origin. ADSCs are now the most studied in the skin, but whether they are the best choice requires additional research and validation. In the future, more mechanism research and large-scale clinical studies are required to establish production or application guidelines for MSCs therapy. To boost the healing capacity of MSCs, the dosage, duration, frequency, and manner of treatment, which have yet to be standardized, should be carefully considered. Therefore, standardized clinical guidelines that can ensure safety and efficacy should be developed before MSCs treatment enters clinical use, so that MSCs can play their maximum role in cosmetic dermatology while being harmless to the human body.

## Data Availability

All data are available on request.

## Declarations

### Footnotes

All statements expressed in this article are solely the author’s statements and do not necessarily represent statements from affiliated organizations, nor do they necessarily represent statements from publishers, editors, and reviewers. Any products that may be evaluated in this article, or any claims that their manufacturers may make, are not guaranteed or recognized by the publisher.

## Abbreviations

MSCs: Mesenchymal stem cells
ESCs: Embryonic stem cells
iPSCs: Induced pluripotent stem cells
ECM: Extracellular matrix
MMP: Matrix metalloproteinase
TGF-β: Transforming growth factor β
UV: Ultraviolet
OS: Oxidative stress
ROS: Reactive oxygen species
HDFs: Human dermal fibroblasts
MAPK: Mitogen-activated protein kinase
AP-1: Activator protein 1
NF-κB: Nuclear factor-κB
α-MSH: α-melanocyte-stimulating hormone
PDGF: Platelet-derived growth factor
IGF-1: Insulin-like growth factor 1
IL-8: Interleukin-8
TNF-α: Tumor necrosis factor-α
ADSCs: Adipose-derived stem cells
BMMSCs: Bone marrow mesenchymal stem cells
UMSCs: Umbilical cord-derived stem cells
EV: Extracellular vesicle
Exo: Exosomes
MV: Microvesicles
MVB: Multivesicular bodies
DPSCs: Dental pulp stem cells
BMP-4: Bone morphogenetic protein 4
SMCs: Smooth muscle cells
VEGF: Vascular endothelial growth factor
HGF: Hepatocyte growth factor
bFGF: Basic fibroblast growth factor
VEGFR2: Vascular endothelial growth factor receptor 2
SMP30: Senescence marker protein 30
IL-1: Interleukin-1
IL-6: Interleukin-6
HaCaTs: Human immortalized keratinocytes
TIMP-1: Tissue inhibitor of metalloproteinases 1
Ang-1: Angiopoietin-1
HIF-1: hypoxia inducible factor-1
CXCL12: C-X-C motif chemokine ligand 12
Nrf2: NF-E2-related factor 2
IFN-γ: Interferon-γ
IL-4: Interleukin-1
IL-10: Interleukin-10
PI3K/Akt: Phosphoinositide 3-kinase/Akt
PTEN: Phosphatase and tensin homolog
SPRY1: Sprouting homologue 1
Nrip1: Nuclear receptor-interacting protein 1
ADSC-CM: ADSC-conditioned medium
HO-1: Heme oxygenase-1
NSC-CM: Neural stem cell-conditioned medium
PDGF-AA: Platelet-derived growth factor AA
3D-HDF-exos: Exosomes formed by three-dimensional growth of HDFs spheres
EGF AgNP: Epithelial growth factor Silver nanoparticle
